# Frontopolar Cortex Interacts With Dorsolateral Prefrontal Cortex to Causally Guide Metacognition

**DOI:** 10.1002/hbm.70146

**Published:** 2025-01-29

**Authors:** Georgia E. Kapetaniou, Marius Moisa, Christian C. Ruff, Philippe N. Tobler, Alexander Soutschek

**Affiliations:** ^1^ Department of Psychology Ludwig Maximilian University Munich Munich Germany; ^2^ Department of Management, Technology, and Economics Swiss Federal Institute of Technology Zurich Zurich Switzerland; ^3^ Department of Economics, Zurich Center for Neuroeconomics University of Zurich Zurich Switzerland; ^4^ Neuroscience Center Zurich, Swiss Federal Institute of Technology Zurich University of Zurich Zurich Switzerland

**Keywords:** frontal pole, intertemporal choice, metacognitive accuracy, theta oscillations, uncertainty

## Abstract

Accurate metacognitive judgments about an individual's performance in a mental task require the brain to have access to representations of the quality and difficulty of first‐order cognitive processes. However, little is known about how accurate metacognitive judgments are implemented in the brain. Here, we combine brain stimulation with functional neuroimaging to determine the neural and psychological mechanisms underlying the frontopolar cortex's (FPC) role in metacognition. Specifically, we evaluate two‐layer neural architectures positing that FPC enables metacognitive judgments by communicating with brain regions encoding first‐order decision difficulty. In support of two‐layer architectures of metacognition, we found that high‐intensity transcranial alternating current stimulation (tACS; 4 mA peak‐to‐peak) over FPC impaired metacognitive accuracy; at the neural level, this impairment was reflected by reduced coupling between FPC and dorsolateral prefrontal cortex (DLPFC), particularly during difficult metacognitive judgments. We also evaluated conceptual accounts assuming that metacognition relies on self‐directed mentalizing. However, we observed no influence of FPC tACS on mentalizing performance and only a weak overlap of the networks underlying metacognition and mentalizing. Together, our findings put the FPC at the center of a two‐layer architecture that enables accurate evaluations of cognitive processes, mainly via the FPC's connectivity with regions encoding first‐level task difficulty, with little contributions from mentalizing‐related processes.

## Graphical abstract

Combining brain stimulation with neuroimaging, we show that the frontopolar cortex causally implements metacognition in value‐based choice by communicating with brain regions involved in first‐order task performance, providing empirical support for theoretical two‐layer accounts of metacognition.

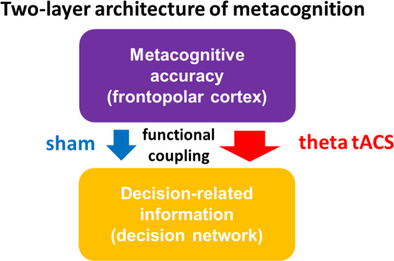

## 1 Introduction

Metacognition is the ability to reliably evaluate and report the accuracy of cognitive processes (Fleming and Lau 2014). In the domain of value‐based choice, for example, metacognitive accuracy refers to the knowledge a decision maker has about the strength of her preference for one choice option over the others (De Martino et al. 2013). Metacognitive capacity strongly affects prospective decision‐making (Soutschek, Bulley, and Wittekind 2022; Soutschek et al. 2021; Soutschek and Tobler 2020) and is particularly important for difficult decisions when agents are unsure about their preferences (Soutschek and Tobler 2020).

At the neural level, the frontopolar cortex (FPC) appears to contribute to accurate metacognitive judgments not only in value‐based choice (De Martino et al. 2013; Soutschek et al. 2021) but also in other domains of cognition like perceptual decisions (Vaccaro and Fleming 2018). However, it seems implausible that the FPC implements this function in isolation, given that it relies on different kinds of decision‐relevant information likely to be represented elsewhere in the brain. For example, decision‐related information such as subjective confidence, decision difficulty, or preference strength is often found to be associated with activation in the frontoparietal control network as well as the neural reward system (Bartra, McGuire, and Kable 2013; Clithero and Rangel 2014; Jimura et al. 2018; Kable and Glimcher 2007; Lebreton et al. 2015; Lin et al. 2018; Rouault, Lebreton, and Pessiglione 2023; Vaccaro and Fleming 2018). Here, we aimed to elucidate the neural and psychological mechanisms underlying the FPC's contribution to metacognition, by empirically testing two (not mutually exclusive) theoretical accounts: two‐neural‐layer architectures and conceptual accounts of metacognition as self‐directed mentalizing. According to two‐layer architectures of metacognition, accurate metacognitive judgments require the FPC to read‐out decision‐related information from cortical regions involved in first‐order task processes (Fleming and Daw 2017; Fleming and Dolan 2012). Correlative evidence indeed suggests that FPC activity is functionally coupled with activity in regions involved in value coding and decision‐making (decision network) during metacognitive evaluations, including lateral and ventromedial prefrontal cortex (VMPFC) (Ainsworth et al. 2022; De Martino et al. 2013). However, it remains unknown whether accurate confidence reports causally require crosstalk between FPC and the decision network, or whether activation in these regions covaries during metacognitive confidence readouts without affecting the resulting judgments. Here, we tested two‐layer accounts of metacognition by investigating the effects of experimentally manipulating FPC activity on both metacognitive judgments and functional interactions with other areas in the decision network.

The second theoretical account we tested focuses more on the psychological function implemented by the FPC during metacognitive judgments. This account captures metacognition as self‐directed mentalizing (Carruthers 2009; Frith 2012; Proust 2007), which is commonly defined as the ability to attribute mental states to others. Mentalizing in false‐belief tasks has been linked to theta oscillations in the prefrontal cortex (PFC) (Yuk, Anagnostou, and Taylor 2020), which at first glance seems to provide biological support for the framework of metacognition as self‐directed mentalizing. In this view, the FPC serves as a common neural basis of both other‐directed and self‐directed mentalizing (i.e., metacognition). Theta oscillations were ascribed a central role in information flow and communication between distant brain regions (Canolty and Knight 2010), making FPC theta oscillations a plausible candidate for integrating information about both one's own and others' cognitive processes. However, meta‐analytic evidence suggests that mentalizing‐ and metacognition‐related activations overlap only in VMPFC and posterior parts of dorsomedial PFC (DMPFC) but not in FPC (Vaccaro and Fleming 2018). Thus, it remains unknown whether the FPC forms a common neural substrate of both mentalizing and metacognition, hampering our conceptual understanding of the nature of metacognitive processes and their relationship to mentalizing.

The goal of this study therefore was to elucidate the causal role of FPC theta oscillations for metacognition, at both neural and psychological levels, by determining the influence of FPC stimulation on network connectivity and mentalizing, respectively. To this end, we combined transcranial alternating current stimulation (tACS) over FPC with functional brain imaging (fMRI). We used 5 Hz theta stimulation to entrain the corresponding oscillations in FPC because they have been linked to metacognitive accuracy (Soutschek et al. 2021; Wokke, Cleeremans, and Ridderinkhof 2017), using 10 Hz alpha tACS as control frequency (Wokke, Cleeremans, and Ridderinkhof 2017). This allowed us to investigate entrainment‐induced changes in the functional connectivity of the FPC with the decision network during metacognitive judgments. We hypothesized that theta tACS over FPC increases metacognitive accuracy during value‐based decisions. If two‐layer architectures hold, then this stimulation‐induced behavioral change should be mirrored on the neural level by stimulation‐induced enhanced functional coupling between FPC and regions thought to belong to the neural decision network (which encodes information about first‐level task performance). Enhancing FPC theta oscillation may thus improve the access to information about first‐order task performance encoded in the decision network via strengthening synchronous firing in the theta range in these regions. Moreover, if the self‐directed mentalizing account holds, then FPC stimulation should affect not only metacognition but also mentalizing, and have corresponding effects on the brain networks underlying both metacognition and mentalizing. Thus, a common function of FPC for metacognition and mentalizing would provide neural support for theoretical approaches conceiving metacognition as self‐directed mentalizing. By contrast, distinct effects accompanied by connectivity in the decision network would support the notion that the FPC is instead more involved in the read‐out of first‐order task‐related information.

## 2 Materials and Methods

### 2.1 Participants

Forty‐one volunteers (mean age = 24.1, range = 18–32, SD = 3.7, 18 females, all right‐handed) who were recruited through the participant pool of the Laboratory for Social and Neural Systems Research at the University of Zurich participated in the experiment. The sample size was determined with an apriori power analysis (alpha = 5%, power = 80%) based on the effect size of Cohen's *d* = 0.54 for the impact of theta tACS on metacognitive sensitivity in our previous study (Soutschek et al. 2021). The power analysis indicated a sample size of 30 participants, which we increased to further increase the power of our design. Data from two participants were excluded due to a lack of variance in confidence ratings, resulting in a final dataset of 39 participants. All participants provided written informed consent and none had a history of neurological or psychiatric disorders. The study and all procedures were approved by the Cantonal Ethics Committee in Zurich. Participants received a compensation of 120 Swiss francs plus a bonus depending on the decisions they made in the experiment.

We collected a further sample of 30 participants (mean age = 24.6, range = 19–33, SD = 3.8, 15 females, all right‐handed) to assess the influence of lower intensity (2 mA) high‐definition (HD) tACS on metacognitive accuracy (without neuroimaging). Three participants showed no variation in their confidence ratings and had to be excluded. This sample was recruited via the participant pool of the Melessa lab at the Ludwig Maximilian University Munich. Again, participants gave written informed consent and had no history of neurological or psychiatric disorders. The study was approved by the ethics committee of the psychology department at Ludwig Maximilian University.

### 2.2 Stimuli and Task Design

Participants performed two tasks in the scanner: a confidence accuracy task and a mentalizing (false‐belief) task. Both tasks were programmed in Matlab (Cogent toolbox).

#### 2.2.1 Confidence Accuracy Task

**FIGURE 1:**
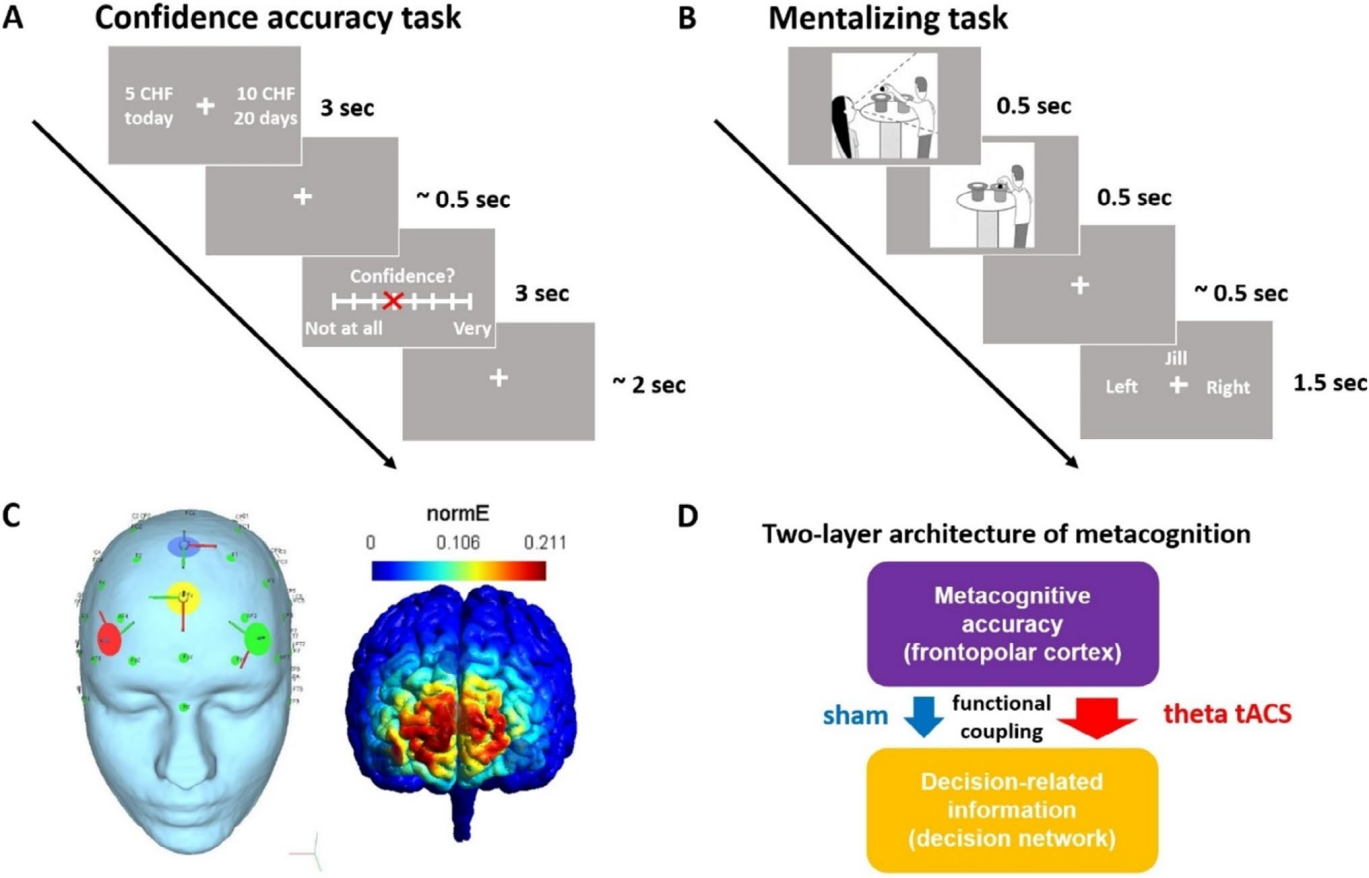
Task design and stimulation setup. (A) In the confidence accuracy task, participants chose between an immediately available smaller‐sooner reward (e.g., 5 CHF today) and a larger‐later reward (e.g., 10 CHF in 20 days). After each decision, they were asked to indicate their confidence in having made the best choice on a scale from 0 to 7. (B) In the mentalizing (false‐belief) task, participants first viewed two consecutive images: in the first image, a person named Jack was holding a ball above one of two hats, while another person named Jill was watching. In the second image, Jack placed the ball in one of the two hats while Jill was absent. Participants had to indicate the position of the ball from either their own or Jill's perspective. Jill held either a true belief (position did not switch) or a false belief (position switched). Participants performed the confidence accuracy and mentalizing tasks in the MRI scanner in an interleaved way. (C) During task performance in the scanner, participants received theta (5 Hz), alpha (10 Hz), or sham tACS using a 3 × 1 electrode setup over the FPC. We estimated the electric field density (normE = volts per meter, V/m) with Simnibs 2.1 (current strength of 4 mA at central electrode). Warmer colors indicate higher electric field density. (D) According to two‐layer accounts of metacognition, the FPC enables accurate metacognitive judgments by reading‐out decision‐related information from other brain regions involved in decision‐making. This predicts that strengthening FPC theta oscillations via FPC tACS should enhance the functional coupling between FPC and the decision network during metacognitive judgments.

Participants performed a monetary intertemporal choice task (also known as delay discounting task) with confidence ratings after each decision (Soutschek, Bulley, and Wittekind 2022; Soutschek et al. 2021). In each trial, participants chose between two monetary rewards, an immediately available smaller‐sooner (SS) reward and a larger‐later (LL) reward. The amounts of the SS reward ranged from 3 to 9 Swiss francs in steps of 1, whereas the LL reward was fixed at 10 Swiss francs which were delivered after a variable delay ranging from 1 to 180 days (1, 2, 5, 10, 20, 40, 60, 90, 120, 180). The two options were presented randomly on the left or right side of the screen in the scanner and participants made their choices by pressing the left or right key, respectively, on an MRI‐compatible response button box. Participants had 3 s to make their choices and received visual feedback on their response (chosen option turned red) for the remaining time of 3 s. Following each choice, participants viewed the fixation cross for a mean of 0.5 s jittered with a Poisson distribution (mean = 0.5 s, range = 0.1–2.0 s) before they had to indicate their confidence in having made the best choice on a rating scale ranging from 0 (not confident at all) to 7 (very confident) within 3 s (Figure 1A). The jitter allowed us to disentangle BOLD signal changes during the decision phase and the confidence ratings. Participants navigated the confidence scale and confirmed their answers using the respective buttons on the response button box. The next trial started after a variable intertrial interval with a mean duration of 2 s and a range of 0.8 to 3.2 s. At the end of the experiment, one trial was randomly selected and implemented. If the participant had chosen an immediate reward in that trial, the corresponding amount was added as a bonus to their compensation, whereas if the participant had chosen the delayed option, 10 Swiss francs were sent to them by mail after the corresponding delay. Participants were instructed about this procedure before performing the task.

In the low‐intensity sample, participants performed the same intertemporal choice task but the currency was Euros instead of Swiss francs and the amounts halved to account for differences in purchasing power. The task was programmed in zTree and post‐decision confidence ratings were given on a continuous sliding window, which we transformed to a scale of 0–7 as in the tACS‐fMRI experiment. In addition to the intertemporal choice task, participants performed a prisoner's dilemma game following previous procedures (Kapetaniou, Deroy, and Soutschek 2023). The results of this task will be reported in a separate manuscript.

#### 2.2.2 Mentalizing Task

We adapted a false‐belief task (Yuk, Anagnostou, and Taylor 2020) in which participants had to indicate the position of a ball either from their own or from another agent's perspective. Each trial started with the presentation of two images. In the first image (0.5 s), an agent named Jack held a ball over one of two hats, while another person called Jill was watching. In the next image (0.5 s), Jack placed the ball in one of the two hats while Jill was absent. The ball either changed position or ended up in the same hat as in the first picture. After a jittered interval (mean = 0.5 s, range = 0.1–2.0 s) during which only the fixation cross was present, the decision screen appeared for 1.5 s together with a cue instructing participants to indicate the ball's position either from their own or from Jill's perspective (Figure 1A). Thus, the task followed a 2 (perspective: Self vs. Jill) × 2 (position: switch vs. no‐switch) design. In the Jill‐perspective condition, participants had to decide whether Jill believed the ball to be in the left or the right hat. If the ball ended up in the same hat as before (no‐switch), Jill held a true belief, whereas if Jack changed the ball's position (switch) Jill held a false belief in the position of the ball. In the self‐perspective condition, participants had to indicate whether or not the ball's position had changed. As a measure of mentalizing demands, we determined performance decrements (accuracy or decision times) when participants had to indicate the ball position after switches versus no switches from Jill's compared to participants' own perspective. After participants provided their response, the chosen option turned red for the remaining time of the 1.5 s. The trial concluded with an intertrial interval whose duration was sampled from a distribution with mean = 2 s and range = 0.8–3.2 s. All positions of the ball, screen sides of the agents, and decision options were counterbalanced across trials.

### 2.3 Procedure

The confidence accuracy task included 180 trials, 60 for each tACS condition. In our previous study, we showed that this trial number is sufficient to reliably detect stimulation effects on metacognition (Soutschek et al. 2021). The mentalizing (false‐belief) task included 216 trials, 72 per tACS condition. Participants performed the tasks in an interleaved design, in six runs of approximately 9 min each (Figure 1A). Each run included 6 miniblocks of 11 trials, including five confidence accuracy trials and six false‐belief trials. The order of the tasks and the stimulation conditions were counterbalanced across participants.

Each block with active stimulation (theta or alpha) started with a 17.5 s stimulation period (including ramp‐up for 1 s) where only the fixation cross was presented on the screen and the participant was instructed to rest. Before the task started, the fixation cross changed color (colors were counterbalanced across participants), indicating the upcoming task. Then, participants performed two miniblocks with trials for the confidence accuracy and the mentalizing task. Each miniblock lasted 72.5 s and was followed by 3 s of rest and two questions (3 s each) where participants rated the stimulation‐induced discomfort and flickering on scales ranging from 0 (not at all) to 7 (very much). The task miniblocks within a stimulation block were separated by a task‐free break of 8.5 s. Thus, a total active stimulation block lasted for 189 s. At the end of the block, the current was ramped down for 1 s. Blocks for sham stimulation included either two or only one task miniblock (contrary to active stimulation blocks which always included two task miniblocks with alpha or theta stimulation in order to minimize total stimulation duration as well as aftereffects of one stimulation type confounding the other). This resulted in three run types, type 1: R—R—S—S—R—R, type 2: R—R—S—R—R—S, type 3: S—R—R—S—R—R, where R corresponds to real (theta or alpha) stimulation and S to sham. If sham was the first stimulation condition, then the sham block lasted for 99 s, starting with 17.5 s fixation and followed by a 72.5 s task miniblock, 3 s rest and 6 s of the stimulation‐induced sensation questions. If sham was not the first stimulation condition, then the sham block started 8.5 s fixation and lasted for 90 s including one task miniblock (8.5 s fixation, 72.5 s task miniblock, 3 s rest, 6 s discomfort and flickering questions), or 180 s including two task miniblocks. Sham blocks were composed of 2 s stimulation intervals (1 s ramp‐up, 1 s ramp‐down; either at theta or alpha frequency), followed by 25 s without stimulation. This pattern was repeated throughout the miniblock to ensure that the sensations of sham matched real stimulation and that participants could not differentiate between the conditions. In line with previous procedures (Moisa et al. 2016; Soutschek et al. 2021), we kept the duration of each stimulation miniblock rather short to minimize stimulation‐induced aftereffects.

Prior to the experimental session, participants filled in an online screening questionnaire to ensure that their time preferences were not extreme and underwent further screening of stimulation and MRI safety criteria. On the main testing day, we applied a local anesthetic paste (Emla Crème 5%, Aspen Pharma Switzerland) to minimize local sensations under the electrodes before the participant entered the scanner. They waited for 45 min for the crème to take effect and we subsequently attached the tACS electrodes.

Next, participants practiced both tasks outside the MRI scanner. They also filled in the short form of the Metacognitions Questionnaire (MCQ‐30; Wells and Cartwright‐Hatton 2004) and the Interactive Mentalizing Questionnaire (Wu, Fung, and Mobbs 2021). At the end of the experiment, they filled in a short demographics questionnaire where they could report potential side effects of the stimulation. There were no serious side effects (reported or occurred).

### 2.4 tACS Protocol

We applied tACS using an 8‐channel tDCS stimulator (DC‐Stimulator MC, neuroConn, Ilmenau, Germany). The active electrode was placed at position AFz according to the international 10–20 system and the three reference electrodes were placed equidistantly (at a distance of 5 cm) around the central electrode forming a triangle (Figure 1C). We used round HD rubber electrodes with diameters of 2 cm. We attached the electrodes using the Ten20 EEG conductive paste (Weaver and Company) and fixated them with bandages throughout the session. Electric field simulations using the Simnibs 2.1 toolbox (Saturnino et al. 2019) suggested that this setup results in a pronounced electrical field in the FPC (Figure 1C). For the stimulation, the tDCS device was placed outside the scanner room and connected to the electrodes with MR‐compatible cables and filter modules. We previously showed that this setup leads to only negligible stimulation effects on fMRI image quality (Moisa et al. 2016). We applied tACS at the theta frequency (5 Hz), alpha as control frequency (10 Hz), and sham at an intensity of 4 mA peak‐to‐peak. For sham tACS, the current was ramped‐up to a maximum intensity of 4 mA and ramped down before the start of the experimental tasks (durations of ramp‐up and ramp‐down phases: 1 s). If participants reported that they could not tolerate an intensity of 4 mA in a pre‐test outside the scanner, we used the highest intensity a participant could tolerate (minimum: 2.4 mA, mean = 3.6 mA). Participants reported no adverse effects of the stimulation during MRI scanning. We refer to the 4 mA stimulation as high‐intensity tACS because it involves a stronger electrical current than the 1.0–2.0 mA that is more common in the field. The stimulation was synchronized with the tasks and fMRI acquisition using a custom‐written software toolbox programmed in Matlab. In the lower‐intensity sample, the tACS setup was identical, but the stimulation intensity was the same (2 mA peak‐to‐peak) for all participants.

### 2.5 MRI Protocol

The neuroimaging data were collected with a Philips Achieva 3‐Tesla whole‐body MR scanner equipped with a 32‐channel standard MR head coil (Philips, Amsterdam, The Netherlands). During each experimental run, we collected 258 volumes with the following parameters: voxel size 3 × 3 × 3 mm^3^, slice gap 0.5 mm, acquisition matrix size 80 × 78, TR/TE = 2334/30 ms, and flip angle 90°. The 40 slices of each volume covered the full brain and were acquired in ascending order. We additionally acquired T1‐weighted multislice fast‐field echo B0 scans to correct for possible field distortions with the following parameters: voxel size 3 × 3 × 3 mm^3^; slice gap 0 mm; acquisition matrix size 80 × 80; TR/TE1/TE2 = 1150/4.6/6.9 ms; flip angle 72°, 50 slices interleaved. Finally, for each participant, we acquired a high‐resolution T1‐weighted 3D fast‐field echo structural scan, which we used during preprocessing for image registration with the following parameters: voxel size 1 × 1 × 1 mm^3^, acquisition matrix size 256 × 256, 170 sagittal slices.

### 2.6 Statistical Analyses

#### 2.6.1 Behavioral Data Analysis

In both the main and the low‐intensity experiment, we estimated each participant's temporal discount factor (separately for each tACS condition) by fitting a hyperbolic discount function to their intertemporal choices in the confidence accuracy task with the hBayesDM package (Ahn, Haines, and Zhang 2017) in R (R Core Team 2020). In hyperbolic discounting, the subjective value (SV) of an LL reward is given by

(1) $$\;{\mathrm{SV}}_{\mathrm{LL}}=\frac{\text{reward magnitude}}{1+k\times \text{delay}.}$$

where *k* corresponds to the individual discounting rate, indicating how strongly the value of future rewards declines as a function of delay (Laibson 1997). This SV of the reward was translated to binary choices by fitting choices with a standard softmax function, which described the probability of the participant selecting the LL reward as a function of the difference in SV between the LL and SS rewards:

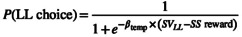

where the *β* parameter (inverse temperature) describes how strongly participants' choices rely on the difference in SVs (choice consistency). For parameter estimation, we used the default options of the hBayesDM toolbox (4 chains with 2000 iterations, burn‐in = 1000). The model was estimated with four Markov Chain Monte Carlo sampling chains of 5000 iterations (1000 warm‐up iterations).

To measure participants' metacognitive access to their preferences, we computed the difference in SV between the two options by subtracting the value of the SS reward from the SV of the LL reward as calculated for each trial using the hyperbolic discounting model and the individual parameter estimates. We analyzed the behavioral data with generalized mixed linear models (GLMMs) in R using the lme4 package (Bates et al. 2014). We regressed choices in the confidence accuracy task (1 = LL, 0 = SS) on fixed‐effects predictors for tACS condition, SV difference (DV_signed_; SV_LL_ minus SV_SS_), confidence rating, and their interactions. As in our previous studies (Soutschek, Bulley, and Wittekind 2022; Soutschek et al. 2021), we z‐standardized confidence and DV_signed_ on the individual level and separately for each tACS condition to control for influences of individual differences in metacognitive bias (confidence) and choice difficulty (DV_signed_), respectively, on metacognitive accuracy. This is necessary because measures of metacognitive accuracy can be confounded with differences in confidence and task difficulty (Fleming and Lau 2014). All fixed‐effect predictors were also modeled as random slopes in addition to participant‐specific random intercepts. We further included predictors for previous tACS, stimulation intensity, stimulation‐related discomfort, and flickering sensations as control variables. As in previous studies (De Martino et al. 2013; Soutschek et al. 2021), we operationalized metacognitive accuracy as the interaction between SV difference and confidence, which quantifies participants' insight into the decision process via the strength of the relationship between revealed decision uncertainty (influence of DV_signed_ on binary choices) and self‐reported decision uncertainty (confidence ratings). The three‐way interaction between tACS, confidence, and DV_signed_ therefore indicates the influence of tACS on metacognitive accuracy. We note that it is not possible to compute computational measures of metacognition like M‐ratio for value‐based decisions because—contrary to e.g. perceptual decisions—one cannot categorize value‐based decisions as correct or incorrect, which is required for computing M‐ratio (Maniscalco and Lau 2012).

For the mentalizing task, we regressed log‐transformed decision times on fixed‐effects predictors for tACS (theta vs. sham and alpha vs. sham), perspective (self vs. Jill), position (switch vs. no‐switch), and all interaction terms. Again, we included discomfort and flickering ratings as well as the tACS condition in the previous block as covariates of no interest. All fixed‐effect predictors were also modeled as random slopes in addition to participant‐specific random intercepts.

#### 2.6.2 MRI Data Analysis

We analyzed fMRI data using statistical parametric mapping (SPM12; www.fil.ion.ucl.ac.uk/spm) in Matlab 2019b (MathWorks). In preprocessing, functional images were motion‐corrected and unwarped. The raw functional, structural, and fieldmap files were reconstructed into a single‐phase file, which was subsequently used to realign and unwarp the functional EPI images. We then performed slice‐timing correction to the middle image and registered the structural images to the mean EPI images. Finally, we performed segmentation and normalization of the images into standard MNI space. Smoothing was performed with an 8 mm FWHM Gaussian kernel and data were further high‐pass filtered with a cutoff of 128 s.

For the first‐level analysis, we computed for each participant a general linear model (GLM‐1) that included onset regressors for the decision period in the intertemporal choice task (duration = decision time) and for the subsequent confidence ratings (duration = time until confirmation of confidence rating), with different onset regressors for sham, theta, and alpha tACS trials. The onset regressor for the decision screen was modulated by a parametric modulator for the absolute SV difference between the LL and SS option (corresponding to the ease of the decision). The onset regressor for the confidence rating screen was modulated by the trial‐specific confidence ratings. The mean correlation between the parametric modulators for confidence and DV_unsigned_ was *r* = 0.34, and variance inflation factors (VIF) as indicators of multicollinearity (confidence: mean VIF = 1.3; DV_unsigned_: mean VIF = 1.2) provided no evidence for problematic mulitcollinearity. We also modeled onset regressors for the four trial types in the mentalizing task (self_switch, self_no switch, Jill_switch, Jill_no switch). All regressors for the intertemporal choice task and the mentalizing task were modeled separately for the three tACS conditions. GLM‐1 further included onset regressors of no interest for response omission trials, discomfort, flickering ratings, and six motion parameters. All regressors were convolved with the canonical hemodynamic response function as implemented in SPM. We computed contrast images for the neural correlated of the absolute value difference and confidence ratings under sham, theta versus sham tACS, and alpha versus sham tACS.

For group‐level analyses, we entered contrast images from the first‐level GLM to random‐effects second‐level analyses to obtain statistical parametric maps. For tests of significance in all whole‐brain analyses, we used cluster‐level inference (cluster‐forming threshold *p* < 0.001 uncorrected) with a statistical threshold of *p* = 0.05 FWE‐corrected for multiple comparisons. The inference was implemented in nonparametric second‐level analyses (computed with the SnPM toolbox (Holmes et al. 1996)) to avoid the risk of inflated false‐positive rates in parametric analyses (Eklund, Nichols, and Knutsson 2016). We conducted an overlap analysis for the neural correlates of confidence and DV_unsigned_ by using the regions negatively correlating with confidence as an explicit mask for DV_unsigned_ on the second‐level. To extract parameter estimates from regions‐of‐interest (ROIs) for visualization and further analyses, we used the Marsbar toolbox (Brett et al. 2002). The ROIs were also used to correct for multiple comparisons within these ROIs via small‐volume correction with FWE correction at peak level (alpha threshold = 5%).

We also conducted a whole‐brain psychophysiological interaction analysis (PPI) to identify brain regions that were functionally connected with FPC during metacognitive judgments and to assess how the FPC's connectivity with these regions was modulated by tACS. Using the routines implemented in SPM12, we extracted the physiological average time series in the FPC seed region and convolved it with confidence and DV_unsigned_ as psychological terms. The FPC seed region (sphere with a diameter of 8 mm (size of kernel filter) centered at *x* = 28, *y* = 50, *z* = 26) was based on a meta‐analysis of the neural correlates of metacognition (Vaccaro and Fleming 2018). As a robustness check, we performed the PPI analysis also with an alternative seed region for Brodmann area 10 from the WFU pickatlas in SPM. We included separate PPI regressors for sham, theta, and alpha tACS. We again performed second‐level analysis in order to derive group‐level inferences on FPC connectivity during metacognitive evaluation.

## 3 Results

### 3.1 FPC tACS Changes Metacognitive Accuracy

**FIGURE 2:**
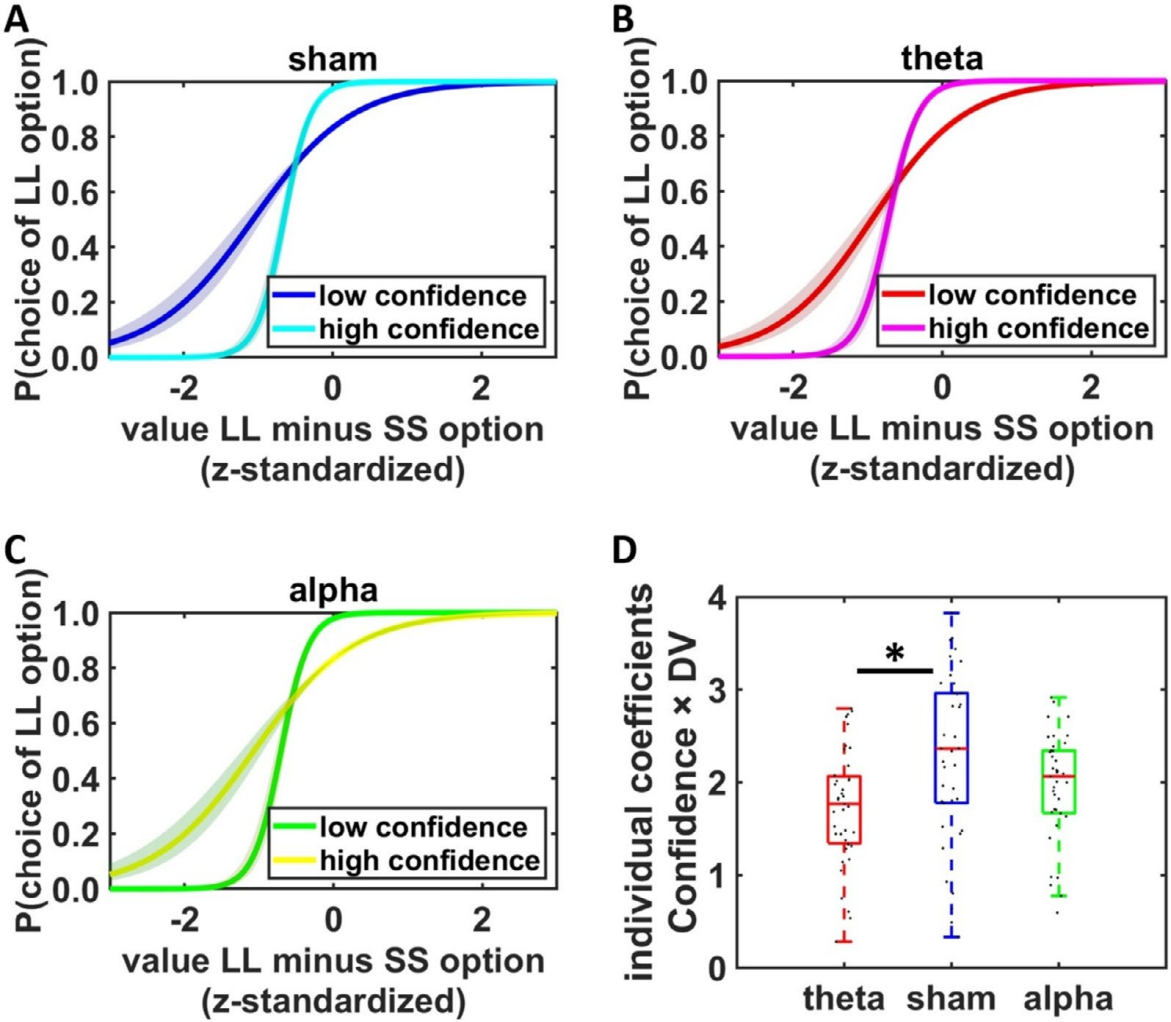
Stimulation effects on metacognitive accuracy. Compared with sham (A), high‐intensity theta tACS (B) but not high‐intensity alpha tACS (C) significantly impaired metacognitive accuracy, indicated by smaller differences between the slopes of logistic curves (which capture revealed decision uncertainty) for low and high confidence decisions. For illustration purposes (not for statistical analysis), we split data into low‐ and high‐confidence decisions. (D) Individual parameter estimates (extracted from the GLMM) for the confidence × DV_signed_ interaction, plotted separated for the tACS conditions. Black dots indicate individual data points. Asterisks indicate significant effects (*p < 0.05).

First, we assessed whether the confidence accuracy task worked similarly as in previous research (Bulley et al. 2022; Soutschek, Bulley, and Wittekind 2022; Soutschek et al. 2021). To do so, we used a GLMM to regress LL versus SS choices on predictors for tACS (theta vs. sham and alpha vs. sham), confidence, and signed value difference (LL minus SS reward value, as given by individually determined hyperbolic discount functions; DV_signed_). As expected, larger value differences were associated with a higher likelihood of selecting the LL reward, beta = 3.99, *z* = 11.01, *p* < 0.001, indicating that participants were sensitive to value. As increasing differences in SVs are associated with decreasing decision uncertainty, the influence of DV_signed_ on binary choices can be interpreted as an indicator of revealed decision uncertainty. Next, we assessed the interaction between confidence and DV_signed_, which indicates the magnitude of the relationship between participants' self‐reported confidence reports and revealed decision uncertainty. Individuals with high metacognitive accuracy should report high confidence when decision uncertainty is low (large positive or negative value differences) and low confidence when decision uncertainty is high (small value differences). The confidence × DV_signed_ interaction thus quantifies the degree to which participants have insight into their decision process, which represents an indicator of metacognitive accuracy (De Martino et al. 2013; Soutschek et al. 2021). This interaction was significant, beta = 2.34, *z* = 8.60, *p* < 0.001, suggesting that participants possessed metacognitive insight into the uncertainty in their decision process. While alpha tACS did not affect metacognitive accuracy compared with sham, beta = −0.37, *z* = 1.31, *p* = 0.18, we observed that theta tACS reduced metacognitive accuracy, beta = −0.55, *z* = 2.08, *p* = 0.04 (Figure 2A–D and Table 1), contrary to our hypothesis and previous findings from our lab of theta FPC tACS increasing metacognitive accuracy (Soutschek et al. 2021). A separate GLMM that tested specifically for differences between theta and alpha tACS revealed no differences in metacognitive accuracy between the effects of theta and alpha tACS, beta = 0.20, *z* = 0.81, *p* = 0.42. The inhibitory theta effect was robust to adding individual mean choices of LL rewards to control for first‐order task performance, beta = −0.57, *z* = 2.45, *p* = 0.02, which has previously been shown to affect metacognitive accuracy (Fleming and Lau 2014; Maniscalco and Lau 2012). The effect was also robust to standardizing confidence ratings on the participant‐specific level to control for individual differences in metacognitive bias, beta = −0.66, *z* = 2.15, *p* = 0.03. We note that the tACS conditions showed no significant differences between log‐transformed discount factors, *F*(1, 36) = 0.06, *p* = 0.81, inverse temperature parameters, *F*(1, 36) = 0.61, *p* = 0.44, on mean confidence ratings, both *t* < 1.40, both *p* > 0.16, or on intraindividual variability in confidence ratings, both *t* < 1.78, both *p* > 0.08. These data provide little support for the notion that the observed effects are related to influences of tACS on first‐order task performance or confidence ratings. Thus, the administered stimulation protocol resulted in reduced metacognitive accuracy under theta tACS.

**TABLE 1:** Results of GLMM explaining choices (1 = LL reward, 0 = SS reward) in the confidence accuracy task. Standard errors of the mean (SE) are in brackets.

Regressor	Beta (SE)	*z*‐value	*p*
Intercept	3.95 (1.58)	2.49	0.01
DV_signed_	3.99 (0.36)	11.01	< 0.001
Confidence	1.24 (0.30)	4.09	< 0.001
tACS_theta_	0.24 (0.32)	0.75	0.45
tACS_alpha_	0.34 (0.34)	0.98	0.33
DV_signed_ × confidence	2.34 (0.27)	8.60	< 0.001
DV_signed_ × tACS_theta_	−0.64 (0.36)	1.76	0.08
DV_signed_ × tACS_alpha_	−0.40 (0.37)	1.07	0.28
Confidence × tACS_theta_	−0.15 (0.20)	0.77	0.44
Confidence × tACS_alpha_	−0.14 (0.20)	0.70	0.48
DV_signed_ × confidence × tACS_theta_	−0.55 (0.27)	2.08	0.04
DV_signed_ × confidence × tACS_alpha_	−0.37 (0.28)	1.31	0.19

There are two potential explanations for the unexpected direction of the stimulation effect: First, we used a more focal HD tACS setup in the current study compared with the less focal electrode setup in our previous study (Soutschek et al. 2021), such that the previous findings might have arisen from stimulation effects on other prefrontal regions than FPC. Second, the current intensity was higher (4 mA for most participants, originally chosen to maximize stimulation effects) in the current than in our previous study (2 mA). The two stimulation intensities may have had reversed effects, as suggested by reports of inverted u‐shaped dose–response relationships (Ehrhardt et al. 2021; Esmaeilpour et al. 2018). To disentangle between these explanations, we collected a further data set where we stimulated the FPC with HD 2 mA tACS, and these results indeed suggest 2 mA tACS to increase metacognitive sensitivity, while 4 mA tACS decreases it (Data S1). We therefore speculate that the inhibitory impact of 4 mA theta FPC tACS on metacognitive accuracy is best explained by an inverted u‐shaped relationship between stimulation intensity and stimulation effects on metacognitive accuracy.

### 3.2 Theta tACS Impairs FPC Coupling With DLPFC During Metacognitive Judgments

**FIGURE 3:**
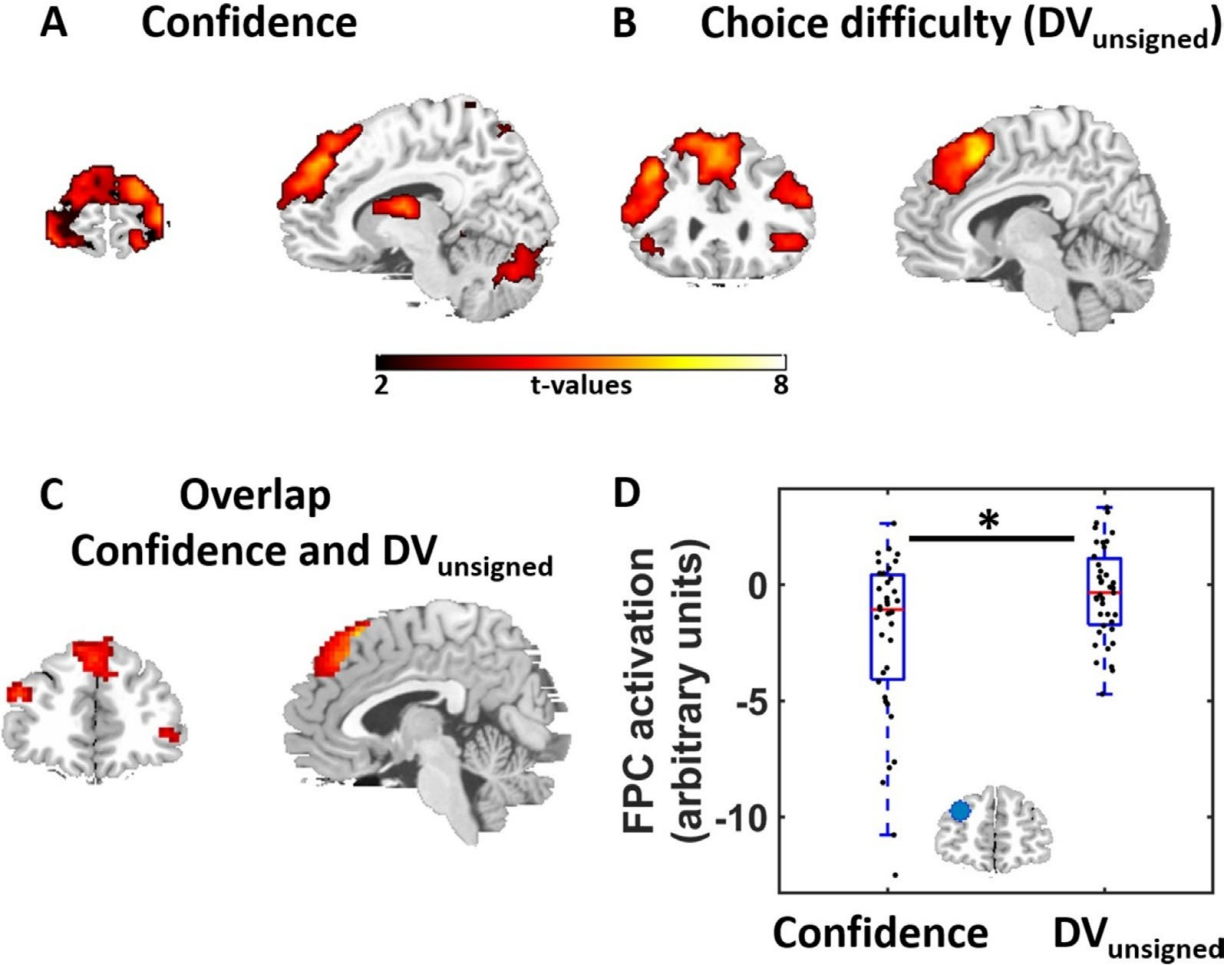
Neural correlates of (*z*‐transformed) confidence and choice difficulty in the sham baseline condition. (A) Confidence ratings negatively correlated with activation in the prefrontal and parietal cortex, including the bilateral FPC. (B) Activations in similar regions correlated with choice difficulty (low values for DV_unsigned_). Activation maps are thresholded at *p* < 0.001 uncorrected, minimum cluster size = 20 voxels. We found no significant tACS effects on the neural correlates of confidence or DV_unsigned_. (C) Overlap between neural correlates of decreasing confidence and choice difficulty. (D) FPC activation was more strongly related to decreasing confidence ratings than to choice difficulty. For illustration purposes, we show here individual parameter estimates extracted from the meta‐analysis FPC ROI (inset). Black dots indicate individual data points. Asterisks indicate significant effects in the second‐level analyses (*p < 0.05).

To obtain a mechanistic understanding of the FPC's role for metacognition, we assessed how FPC‐targeted tACS affected FPC connectivity with brain regions involved in decision‐making. Based on previous findings, we hypothesized the FPC, and in particular FPC theta oscillations (Soutschek et al. 2021), to be functionally connected to the neural decision network, particularly during difficult metacognitive judgments (De Martino et al. 2013). Combining tACS with neuroimaging allowed us to test whether FPC tACS changes metacognitive accuracy by modulating the FPC's connectivity with regions encoding decision‐relevant information. Consistent with previous findings (Vaccaro and Fleming 2018), in the sham condition decreasing confidence ratings (parametric modulator) correlated with stronger activation in several prefrontal and parietal regions (Figure 3 and Table 2), including the FPC. This replicates previous findings of increased FPC activity during low‐confidence metacognitive judgments (De Martino et al. 2013; Fleming, Huijgen, and Dolan 2012; Lebreton et al. 2015), which may reflect the trials when metacognitive judgments are most difficult and demands on metacognitive processes are highest. The activation in the FPC was also significant when we assessed activation selectively for FPC in ROI based on either a meta‐analysis on metacognition (Vaccaro and Fleming 2018), *t*(38) = 3.97, *p* = 0.004, small‐volume corrected, or on an anatomical mask for Brodmann area 10, *t*(38) = 6.98, *p* < 0.001, small‐volume corrected. No brain regions positively correlated with increasing confidence ratings, and exploratory whole‐brain analyses showed no significant influence of theta or alpha tACS on neural correlates of confidence, all *p* > 0.07, FWE‐corrected at cluster level.

**TABLE 2:** Anatomical locations and MNI coordinates of the peak activations correlate negatively with confidence in GLM‐1. We report activations surviving whole‐brain FWE correction at peak or cluster level (*p* < 0.05) and local maxima more than 8 mm apart (default option in SPM12). Note that decreasing confidence correlated with a large cluster with peaks in FPC that extended to bilateral and dorsomedial prefrontal cortex.

Region	MNI coordinates						
	Hem	BA	*X*	*Y*	*Z*	*k*	*T*
Cerebellum	L		−27	−79	−34	1145	7.63
Frontopolar cortex	L	10	−42	56	−1	8639	6.98
Temporal gyrus	R	21	66	−40	−7	1409	6.51

Abbreviations: BA = brodmann area; Hem = hemisphere (L = left, R = right).

Increasing choice difficulty (i.e., decreasing values of DV_unsigned_) was associated with activation in a network including DLPFC, dorsomedial PFC, and parietal cortex (whole‐brain analysis; Figure 3B and Table 3), regions that have been assigned to the decision network (Wesley and Bickel 2014). Choice difficulty showed strong overlap with the networks underlying confidence judgments in an overlap analysis (Figure 3C). Moreover, choice difficulty correlated with FPC activation only within the anatomical FPC ROI, *t*(38) = 4.63, *p* = 0.02, small‐volume corrected, but there were no suprathreshold voxels within the meta‐analysis‐based FPC ROI. When we directly compared FPC activity related to confidence versus DV_unsigned_, we observed that FPC activity was more strongly associated with decreasing confidence ratings than with choice difficulty both in the meta‐analysis‐based and the anatomical mask, both *t*(38) > 2.48, both *p* < 0.02 (Figure 3D). Theta or alpha tACS had no influence on neural correlates of choice difficulty, all *p* > 0.18, FWE‐corrected at the cluster level. Moreover, second‐level correlation analyses provided no evidence that stimulation effects on confidence or DV_signed_ (from the behavioral GLMM) correlated with the corresponding tACS effects on the neural level, all *p* > 0.72, FWE‐corrected at the cluster level. This suggests that the stimulation did not affect the processing of decision‐relevant information per se. Taken together, local FPC activity was associated with making difficult metacognitive judgments, whereas choice difficulty was encoded in a frontoparietal network.

**TABLE 3:** Anatomical locations and MNI coordinates of the peak activations correlating negatively with DV_unsigned_ (i.e., correlating positively with choice difficulty) in GLM‐1. We report activations surviving whole‐brain FWE correction at peak or cluster level (*p* < 0.05).

Region	MNI coordinates						
	Hem	BA	*X*	*Y*	*Z*	*k*	*T*
Dorsomedial and dorsolateral prefrontal cortex	L/R	8	0	20	50	1915	7.13
Angular gyrus	R	40	45	−43	44	655	5.80
Premotor cortex	L	6	−45	8	47	724	5.62
Angular gyrus	L	40	−45	−43	44	101	4.83

Abbreviations: BA = brodmann area; Hem = hemisphere (L = left, R = right).

**FIGURE 4:**
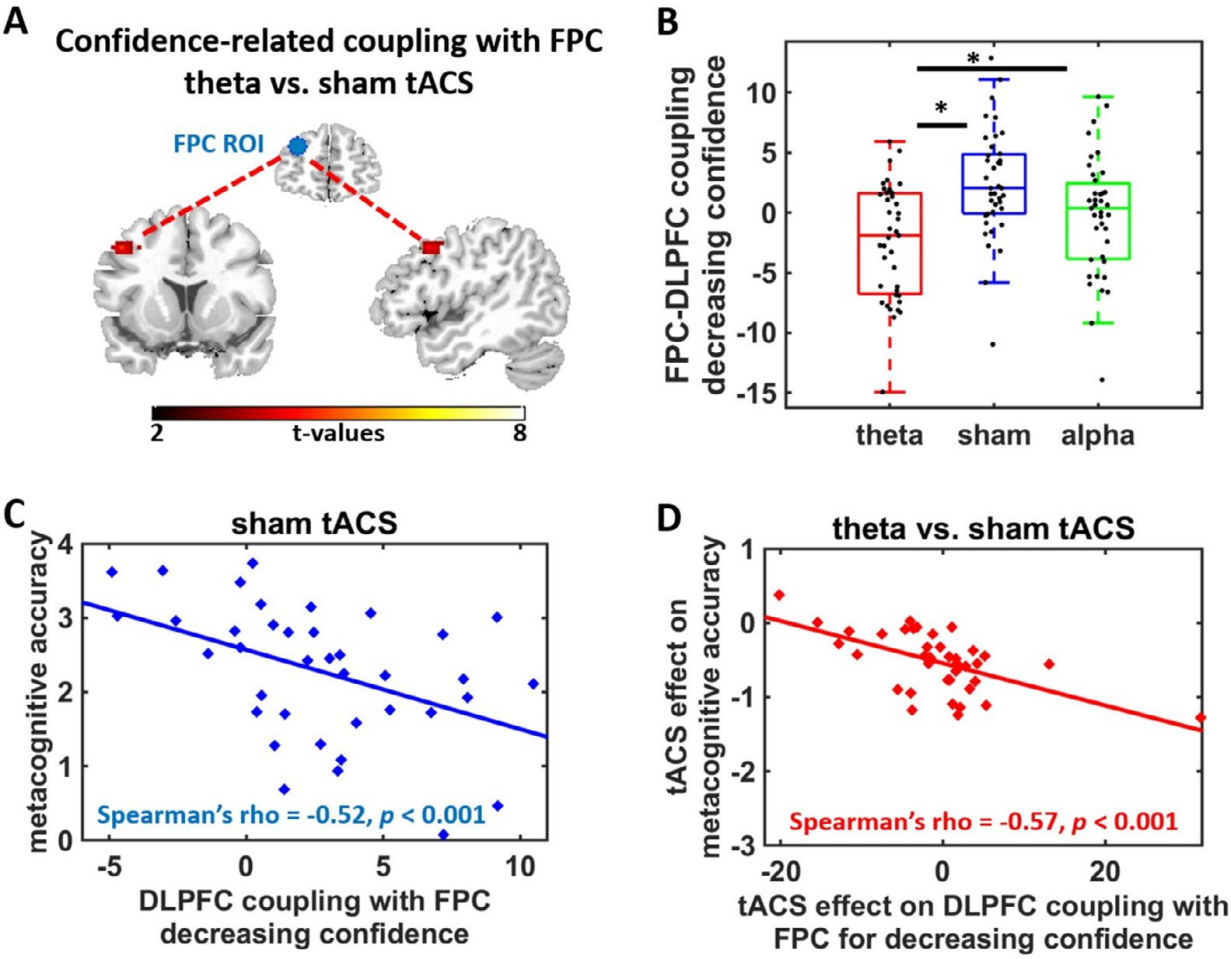
Stimulation effects on functional coupling with FPC. (A) Theta tACS significantly modulated the confidence‐related coupling of DLPFC with FPC (seed region) compared with sham and alpha tACS. (B) Parameters extracted from the significant DLPFC cluster suggest that under sham the FPC shows enhanced coupling with DLPFC for decreasing confidence ratings (i.e., difficult metacognitive judgments), and this enhanced coupling for decreasing confidence trials is reduced under theta tACS. Note that in this plot extracted parameters are for illustration purpose only, not for statistical inference. Asterisks indicate significant effects in the second‐level analyses (*p < 0.05). (C) Under sham, DLPFC coupling with the FPC seed region for decreasing confidence related to individual differences in metacognitive accuracy, and (D) stimulation effects on FPC‐DLPFC coupling correlated with the influence of theta tACS on the accuracy of metacognitive judgments: worse metacognitive skills (either under sham or as result of FPC tACS) were associated with stronger FPC‐DLPFC coupling for decreasing confidence.

According to two‐layer architecture accounts, the FPC implements metacognitive accuracy not in isolation but in interaction with regions encoding decision‐related information. We therefore assessed how tACS changed the functional coupling of FPC with other brain regions. Specifically, we conducted a psychophysiological interaction (PPI) analysis with the FPC ROI from the meta‐analysis on metacognition (Vaccaro and Fleming 2018) as seed region and confidence ratings as well as DV_unsigned_ as separate psychological terms. Consistent with the hypothesized coupling between FPC and the decision network, FPC activity during the sham condition was more functionally coupled with right DLPFC for decreasing confidence (i.e., during more difficult versus easier metacognitive judgments), *t*(38) = 4.31, *p* = 0.03, small‐volume FWE‐corrected within prefrontal mask for neural correlates of choice difficulty (DV_unsigned_, mask thresholded at *p* < 0.001 uncorrected). Thus, FPC communicates with a DLPFC subregion encoding choice difficulty during metacognitive judgments (Figure 4A,B). If this FPC‐DLPFC connectivity underlies metacognitive readouts, it should be linked to behavioral measures of metacognitive accuracy. Correlation analyses supported this: FPC‐DLPFC coupling for decreasing confidence (extracted from the significant DLPFC cluster in the confidence PPI analysis) was significantly correlated with metacognitive accuracy on the behavioral level (individual coefficients for the DV_signed_ × confidence interaction from the behavioral GLMM), *r* = −0.47, *p* = 0.002; nonparametric: Spearman's rho = −0.52, *p* < 0.001; Figure 4C. The negative sign of the correlation is consistent with previous findings (De Martino et al. 2013) and suggests that individuals with weaker metacognitive accuracy on the behavioral level showed stronger FPC‐DLPFC connectivity for decreasing confidence, whereas individuals with better metacognitive skills showed weaker confidence‐related FPC‐DLPFC coupling. Individuals with poorer metacognitive skills may therefore have to reflect harder on the accuracy of their decisions (mirrored by enhanced FPC‐DLPFC connectivity) than individuals with good metacognitive knowledge about their decisions. Taken together, these findings link the accuracy of metacognitive judgments to the strength of FPC‐DLPFC connectivity.

Next, we tested for stimulation effects on this confidence‐related FPC coupling. Theta versus sham tACS significantly reduced the FPC's confidence‐dependent connectivity with right DLPFC, *x* = 45, *y* = 8, *z* = 41, cluster size = 158 voxel, *t*(38) = 4.65, *p* = 0.02, FWE‐corrected at cluster level. This cluster was part of the network encoding choice difficulty, as suggested by an overlap analysis with the neural correlates of choice difficulty under sham. Moreover, the stimulation effects on FPC‐DLPFC connectivity (extracted from the prefrontal choice difficulty ROI) correlated with the impact of tACS on metacognitive accuracy on the behavioral level (individual coefficients for tACS_theta_ × DV_signed_ × confidence), *r* = −0.58, *p* = 0.001, nonparametric: Spearman's rho = −0.57, *p* < 0.001 (Figure 4D). Closer inspection suggested that, in line with the direction of the effect under sham, tACS‐induced impairments in metacognitive accuracy were associated with more negative FPC‐DLPFC coupling under theta compared with sham tACS. There was no evidence for significant effects of alpha tACS on confidence‐dependent connectivity with FPC, all *p* > 0.27, FWE‐corrected at cluster level, and theta tACS showed stronger effects than alpha tACS on confidence‐related FPC coupling in DLPFC, *x* = 27, *y* = 38, *z* = 50, *t*(38) = 4.83, *p* = 0.03, FWE‐corrected at cluster level. Lastly, the FPC showed no connectivity with other regions depending on DV_unsigned_, neither under sham nor for theta versus sham tACS, all *p* > 0.28, FWE‐corrected at cluster level. This suggests the FPC‐DLPFC connectivity to be specific for metacognitive judgments rather than value computation. Taken together, our findings suggest that FPC stimulation affects metacognitive accuracy by modulating the FPC's connectivity with brain regions encoding decision‐related information.

As robustness check, we recomputed the PPI analysis with an alternative FPC seed region based on the anatomical mask for Brodmann area 10. Again, theta versus sham tACS significantly reduced confidence‐related connectivity of FPC with right DLPFC, *x* = 24, *y* = 17, *z* = 56, *t*(38) = 4.85, *p* = 0.01, FWE‐corrected at cluster level, but also with the midcingulate cortex, *x* = −6, *y* = −16, *z* = 38, *t*(38) = 4.26, *p* = 0.01, FWE‐corrected at cluster level. This underlines the robustness of our findings and suggests the FPC to implement accurate metacognitive judgments via functional coupling with a prefrontal network encoding preference strength.

### 3.3 No Evidence for FPC Involvement in Mentalizing

**FIGURE 5:**
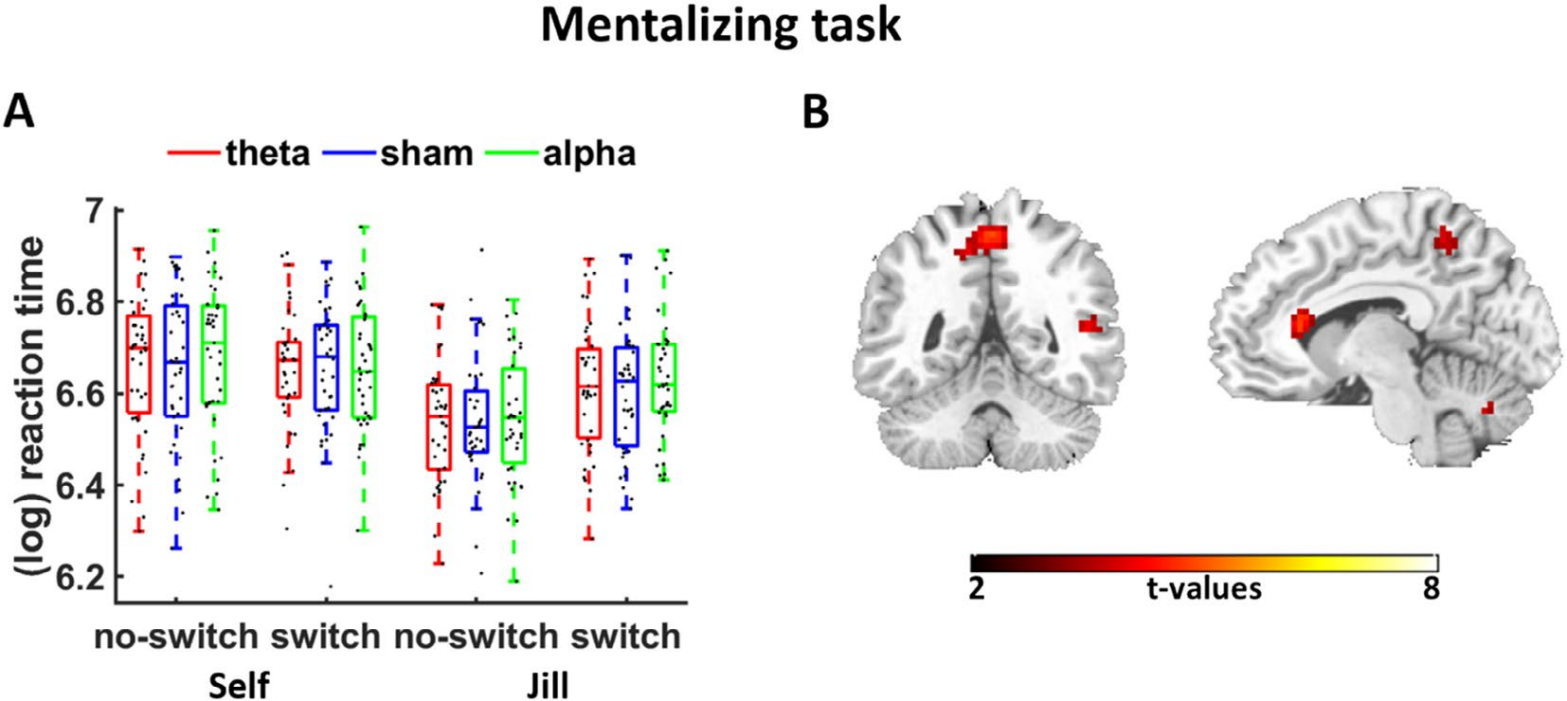
Behavioral and imaging results for the mentalizing (false‐belief) task. (A) FPC theta or alpha tACS did not significantly affect performance (log‐transformed reaction times) in the mentalizing task. (B) Mentalizing demands (Switch>No‐switch)_Jill_ > (Switch>No‐switch)_self_ significantly correlated with activation in regions belonging to the mentalizing network, including precuneus and posterior temporal cortex. Activation maps are thresholded at *p* < 0.001 uncorrected, minimum cluster size = 20 voxel. We found no significant tACS effects on the neural correlates of mentalizing.

Last, we assessed whether the FPC's role for metacognition is related to mentalizing, inspired by theoretical accounts that conceptualize metacognition as self‐directed mentalizing (Proust 2007). In the mentalizing task, participants indicated the position of a ball either from their own or an avatar's (“Jill”) perspective who could not observe switches of the ball position (Yuk, Anagnostou, and Taylor 2020). Correctly reporting the ball position after switches compared to no switches from Jill's perspective (relative to one's own perspective) required mentalizing abilities in order to understand that Jill holds a false belief in the switch condition. In line with this, decision times were slower for switch vs. no‐switch trials particularly in the Jill (rather than the self) condition, perspective × position: beta = 0.06, *t*(45) = 2.3, *p* = 0.03. However, there was no evidence for significant effects of theta tACS, beta = 0.03, *t*(126) = 0.9, *p* = 0.37, or alpha tACS, beta = 0.05, *t*(255) = 1.8, *p* = 0.07, on the perspective × position interaction (Figure 5A and Table 4). Also Bayes factors (computed with the Savage Dickey approach) indicated that the null hypothesis (no stimulation effect) was more likely than the alternative hypothesis (stimulation effects on mentalizing) for both theta, BF_01_ = 24.3, and alpha tACS, BF_01_ = 7.4. Taken together, the data provide no conclusive evidence for FPC involvement in mentalizing (as would be indicated by stimulation effects on the perspective × position interaction), though they also do not clearly favor the null hypothesis of no tACS effects on mentalizing. Finally, when we assessed whether individual variation in mentalizing predicts metacognitive abilities, we found a negative (instead of positive) correlation between metacognition (individual coefficients for confidence × DV_signed_) and mentalizing (coefficients for perspective × position) under sham, *r* = −0.39, *p* = 0.01, providing, if anything, evidence against the “metacognition as mentalizing” account. Moreover, theta tACS effects on metacognition and mentalizing were uncorrelated, *r* = −0.02, *p* = 0.88. On the neural level, we observed no overlap between the neural correlates of confidence and mentalizing (Figure 5B, Data S1). Together, our findings fail to support the hypothesized positive link between metacognition and mentalizing.

**TABLE 4:** Results of GLMM regressing log‐transformed reaction times in the mentalizing task on predictors for tACS, perspective (self vs. Jill), and position (no switch vs. switch). Standard errors of the mean (SE) are in brackets.

Regressor	Beta (SE)	*t*‐value (df)	*p*
Intercept	6.70 (0.03)	232.8 (52)	< 0.001
Perspective	−0.11 (0.02)	5.8 (48)	< 0.001
Position	−0.00 (0.02)	0.1 (52)	0.96
tACS_theta_	−0.03 (0.02)	1.7 (1002)	0.09
tACS_alpha_	−0.02 (0.02)	0.8 (145)	0.41
Perspective × position	0.06 (0.03)	2.3 (45)	0.03
Perspective × tACS_theta_	−0.01 (0.02)	0.4 (296)	0.68
Perspective × tACS_alpha_	−0.02 (0.02)	1.1 (96)	0.27
Position × tACS_theta_	−0.01 (0.02)	0.6 (683)	0.57
Position × tACS_alpha_	−0.03 (0.02)	1.2 (69)	0.24
Perspective × position × tACS_theta_	0.03 (0.03)	0.9 (126)	0.37
Perspective × position × tACS_alpha_	0.05 (0.03)	1.8 (255)	0.07

## 4 Discussion

Accurate metacognitive judgments in value‐based decisions require individuals to evaluate the strength of their preference between choice options. Here, we identify the neural mechanisms enabling decision makers to align their post‐decision confidence with the strength of their economic preferences. The FPC, a region that has been ascribed a central role for metacognitive processes (Vaccaro and Fleming 2018), showed enhanced functional coupling with parts of the DLPFC encoding preference‐related information during difficult metacognitive judgments, with the strength of this connectivity explaining individual variation in metacognitive accuracy. FPC theta tACS significantly reduced FPC‐DLPFC coupling, mirroring the inhibitory stimulation effects on metacognitive accuracy on the behavioral level. The unexpected direction of the stimulation effects on metacognitive accuracy could be explained by an inverted u‐shaped dose–response curve of tDCS (Batsikadze et al. 2013; Ehrhardt et al. 2021; Esmaeilpour et al. 2018; Moliadze et al. 2012). It has been suggested that higher tDCS intensities lead to a non‐optimal balance between excitatory and inhibitory cortical mechanisms (Krause, Marquez‐Ruiz, and Cohen Kadosh 2013). We speculate that this might explain the opposite effects of 4 mA compared with 2 mA tACS on metacognition. We note that in the behavioral analyses we observed no significant difference between theta and alpha tACS (contrary to the imaging analysis where theta tACS significantly modulated FPC‐DLPFC connectivity compared with sham), such that we must be careful with inferences regarding the frequency‐specificity of our findings. In any case, the current data suggest the FPC to link preference strength with self‐reported decision confidence by strengthening functional connectivity with a subregion of the DLPFC that encodes decision‐related information.

Our findings support theoretical accounts assuming a two‐layer architecture of metacognitive processes (Fleming and Daw 2017; Fleming and Dolan 2012; Pasquali, Timmermans, and Cleeremans 2010). According to these accounts, the first‐order network encodes decision‐ and performance‐related information (like preference strength for value‐based choice, or perceptual evidence for perceptual decisions), which is then communicated to the second‐order network for metacognitive confidence assessment. Our data suggest that this second‐order process might be implemented by functional coupling of the FPC with brain regions involved in first‐order task performance like the DLPFC (which in the context of our task encodes choice difficulty). In fact, DLPFC perturbation was shown to impair confidence computations without affecting metacognitive accuracy (Shekhar and Rahnev 2018), supporting the idea of a functional segregation between regions for first‐order and second‐order task processing. The hypothesized involvement of the FPC for second‐order confidence judgments is supported by findings showing the FPC to orchestrate the mapping from private to public confidence reports (Bang et al. 2020), whereas confidence judgments about others' (rather than one's own) perceptual decisions activate the mentalizing network (Bang et al. 2022). We note, though, that the current task design does not allow dissociating between confidence in acting consistently with one's preferences and uncertainty about one's true preferences, two processes that were found to be dissociable and to be represented in distinct brain regions (Bang and Fleming 2018; Pouget, Drugowitsch, and Kepecs 2016).

The read‐out of information from distant brain regions may be linked to neural theta oscillations, given that synchronous firing in the theta frequency has been related to information flow between distant brain networks (Canolty and Knight 2010). We note that we make no claims about the domain generality of FPC‐DLPFC coupling for metacognition in other domains of cognition like perceptual decisions or memory. While the FPC and further prefrontal regions like dorsomedial and dorsolateral PFC were linked to self‐reported confidence across different domains—like value‐based and perceptual decisions (Vaccaro and Fleming 2018)—the neural correlates of first‐order task performance in these domains are at least partially dissociable. Besides the frontoparietal control network, perceptual decisions were linked to areas involved in sensory processing (Heekeren, Marrett, and Ungerleider 2008; Keuken et al. 2014). We therefore assume that the FPC causally implements metacognitive accuracy also in other domains, but by communicating with different neural networks for first‐order task performance. Given the evidence for coupling of FPC with posterior visual areas during metacognitive judgments in perceptual decisions (Fleming, Huijgen, and Dolan 2012), we speculate that the FPC may retrieve performance‐related information from domain‐specific brain regions representing first‐order task performance in a given cognitive domain like sensory regions in perceptual decisions.

Besides determining the neural networks underlying metacognition, we also investigated the FPC's role for metacognition on a psychological level. Here, our results provide little support for conceptual accounts of metacognition as self‐directed mentalizing. If metacognition were just a special case of inferring the mental states of others, we should see an effect of FPC stimulation on mentalizing as well as strong overlap between the networks underlying mentalizing and metacognition. In contrast to these predictions, there was no evidence for stimulation effects on behavioral measures of mentalizing. Moreover, while stronger demands on metacognition were associated with activation in FPC and a frontoparietal network, mentalizing demands correlated with activation in the canonical mentalizing network including precuneus and right temporo‐parietal junction (Schurz et al. 2014; Van Overwalle and Baetens 2009). The weak overlap between these networks, as well as the absence of a significant FPC tACS effect on mentalizing performance, suggest that mentalizing and metacognition rely on distinct neural networks that overlap only in a few regions like precuneus. The weak overlap is consistent with meta‐analytic evidence suggesting dissociable neural networks (except for VMPFC and DMPFC) to correlate with metacognitive and mentalizing processes (Vaccaro and Fleming 2018). Thus, while the lack of substantial overlap between these neural networks cannot directly reject the metacognition‐as‐self‐related‐mentalizing account, it undermines the biological plausibility of this view. We note that we chose metacognition and mentalizing paradigms with very different task demands to avoid the issue that two tasks from a similar domain (e.g., a self‐directed and an other‐directed value‐based choice task) positively correlate due to the shared task demands rather than due to common underlying mechanisms of metacognition and mentalizing. Remarkably, individual differences in metacognitive accuracy correlated negatively, rather than positively, with mentalizing abilities. An ad hoc explanation for this unexpected finding might be that metacognition requires focusing on one's own mental states, whereas mentalizing on the contrary demands disengaging from one's own thoughts in order to adopt the perspective of others. As a caveat, we note that this finding may not necessarily generalize to all other metacognition and mentalizing tasks, so future studies should replicate our results using alternative experimental paradigms. Lastly, it is worth keeping the conceptual differences between mentalizing and metacognition in mind: while metacognition evaluates the accuracy of a cognitive process (i.e., whether the most‐preferred option was chosen), mentalizing assigns mental states to agents without evaluating the accuracy of these mental states. This functional differentiation makes it plausible that a brain network evolved that monitors specifically one's own mental processes.

The FPC is not only phylogenetically young (Fuster 2001) but also rapidly expanded during the hominid evolution (Semendeferi et al. 2001). It has developed in size and connectivity more than any other brain area compared to nonhuman animals, suggesting that it might enable uniquely human functions (Frith 2012). The high density of dendritic spines in FPC (Semendeferi et al. 2011) and its fiber connections to other prefrontal regions reflect the FPC's capacity for integrating information from distant regions at a high level of abstraction (Ramnani and Owen 2004; Velichkovsky et al. 2018), a feature thought to have developed especially in humans (Bludau et al. 2014). Metacognition is hypothesized to have evolved also relatively recently in human evolution, in line with the increased need to express the inferred accuracy of our cognitive processes and share it with others (Frith 2012; Shea et al. 2014). Our findings inform these theoretical accounts by showing that metacognition indeed seems to rely on connections between FPC and lower‐level regions involved in first‐order task performance.

Taken together, our findings provide a network perspective on metacognition, in which the FPC enables accurate metacognitive evaluations of cognitive performance by communicating with brain regions involved in first‐order task performance. By supporting a two‐layer account, our study provides a mechanistic understanding of the brain networks underlying metacognitive accuracy.

## Author Contributions

G.E.K., M.M., C.C.R., P.N.T. and A.S. designed research; G.E.K. and M.M. performed research; G.E.K. and A.S. analyzed data; G.E.K. and A.S. wrote the first draft of the manuscript; all authors approved the final manuscript version.

## Conflicts of Interest

The authors declare no conflicts of interest.

## Supporting information


**Data S1.** Supporting Information.

## Data Availability

The behavioral data supporting the findings of this study are publically available on the Open Science framework (OSF; https://osf.io/dbp34/). Neuroimaging data will be provided by the authors upon reasonable request.
