# Supplementary material for: Frontopolar Cortex Interacts With Dorsolateral Prefrontal Cortex to Causally Guide Metacognition
Source: Hum Brain Mapp. 2025 Jan 29;46(2):e70146. doi: 10.1002/hbm.70146 (PMC11775761; doi:10.1002/hbm.70146)
Supplement: Supplementary file 1 — Data S1. Supporting Information. [file HBM-46-e70146-s001.docx]

**Supporting information for**

**Frontopolar cortex interacts with dorsolateral prefrontal cortex**

**to causally guide metacognition**

Georgia E. Kapetaniou^1,2 †^, Marius Moisa^3 †^, Christian C. Ruff^3,4^, Philippe N. Tobler^3,4^, Alexander Soutschek^1^

^1^ Department of Psychology, Ludwig Maximilian University Munich, Munich, Germany

^2^ Department of Management, Technology, and Economics, Swiss Federal Institute of Technology Zurich, Zurich, Switzerland

^3^ Zurich Center for Neuroeconomics, Department of Economics, University of Zurich, Zurich, Switzerland

^4^ Neuroscience Center Zurich, University of Zurich, Swiss Federal Institute of Technology Zurich, Zurich, Switzerland

**Supporting results**

*Behavioral analyses*

We tested for stimulation effects on decision times in the confidence accuracy task by regressing log-transformed decision times on predictors for tACS, Confidence, DV_unsigned_, and all interaction effects. We observed longer decision times for decreasing confidence, beta = -0.06, *t*(40) = 8.35, *p* < 0.001, as well as for increasing choice difficulty (DV_unsigned_), beta = -0.04, *t*(44) = 5.63, *p* < 0.001. However, there was no evidence for significant effects of theta or alpha tACS on decision times, all *t* < 1.3, all *p* > 0.21.

In a further control analysis, we assessed whether significant effects of theta tACS on metacognitive accuracy are robust to not z-standardizing confidence and DV_signed_ separately for each tACS condition. This analysis revealed no significant effect on theta tACS on the Confidence × DV_signed_ interaction, beta = -0.38, *z* = 1.37, *p* = 0.17. However, this analysis should not be interpreted as evidence against the involvement of FPC theta oscillations in metacognitive accuracy, as it does not control for confounding (non-significant) differences in confidence and choice difficulty between the tACS conditions on metacognitive accuracy.

*Control experiment*

To assess whether the negative effects of 4mA FPC tACS on metacognitive sensitivity can be explained by an inverted u-shaped dose-response curve, we collected a further data set using the current high-definition electrode setup but with a weaker current intensity (2 mA). An analysis over both data sets (again controlling for first-order task performance with mean LL choices as well as for individual differences in metacognitive bias) revealed a significant Experiment × tACS_theta_ × DV_signed_ × Confidence interaction, beta = 1.94, *z* = 2.78, *p* = 0.01, suggesting that 2 mA versus 4 mA theta tACS affected metacognitive accuracy in different directions. There was also evidence for dissociable effects of alpha tACS on metacognition depending on stimulation intensity, Experiment × tACS_alpha_ × DV_signed_ × Confidence: beta = 1.02, *z* = 2.39, *p* = 0.02. To assess whether 2 mA tACS increased rather than reduced metacognitive accuracy, we conducted a separate GLMM on the 2 mA data set, which revealed enhanced metacognitive accuracy under theta tACS, beta =2.61, z = 2.02, p = 0.04, as well as alpha tACS, beta =3.40, z = 2.40, p = 0.02. A separate GLMM revealed no significant differences between theta and alpha: beta = 0.48, z = 0.47, p = 0.64. In any case, based on the comparison between low and high-intensity stimulation conditions, we speculate that the inhibitory impact of 4 mA theta FPC tACS on metacognitive accuracy is best explained by an inverted u-shaped relationship between stimulation intensity and stimulation effects on metacognitive accuracy.

*Neural correlates of mentalizing*

On the neural level, mentalizing demands (Jill_switch>no-switch_ > Self_switch>no-switch_) correlated with activation in precuneus, *p* = 0.02, whole-brain FWE-corrected at cluster level (Figure 5B), a region belonging to the canonical mentalizing network (Schurz, Radua, Aichhorn, Richlan, & Perner, 2014; Vaccaro & Fleming, 2018). A conjunction analysis revealed that the precuneus correlated both with mentalizing demands and confidence judgements, but no voxels overlapped in the prefrontal cortex even at lenient, exploratory thresholds (*p* < 0.001, uncorrected, minimum cluster size = 20 voxel). There were no significant effects of theta or alpha tACS on mentalizing-related neural activity, all *p* > 0.07, FWE-corrected at cluster level. Thus, both the behavioral and the neuroimaging data provide little evidence for FPC involvement in mentalizing.
